# Case report: Cerebrospinal fluid neutrophilic pleocytosis upon intrathecal triamcinolone injection

**DOI:** 10.3389/fneur.2024.1372266

**Published:** 2024-04-23

**Authors:** Thanos Tsaktanis, Jenny Stritzelberger, Vi Tuong Daniel To, Martin Uhl, Stefan Schwab, Dieter Heuss, Veit Rothhammer

**Affiliations:** ^1^Department of Neurology, University Hospital Erlangen, Erlangen, Germany; ^2^Deutsches Zentrum Immuntherapie (DZI), Uniklinikum Erlangen, Erlangen, Germany

**Keywords:** triamcinolone, chemical meningitis, spasticity, side effect, intrathecal injection of triamcinolone, inflammatory CSF syndrome

## Abstract

Intrathecal corticosteroids, initially employed in the 1950s, faced declining use due to complications like arachnoiditis and aseptic meningitis. Triamcinolone, which is nowadays used as intrathecally applied glucocorticoid formulation, has been shown to beneficially influence spasticity without demonstrable influence on disease activity or progression. We here present the case of a patient with recurrent episodes of aseptic cerebrospinal fluid (CSF) neutrophilic pleocytosis over a year following intrathecal triamcinolone treatment. CSF analyses revealed a post-injection CSF cytokine profile resembling cytokine release reaction rather than drug hypersensitivity. This case thus highlights a potential side effect of intrathecal triamcinolone injection with yet unclear clinical relevance, underscores the need for further assessment of clinical benefits of intrathecal triamcinolone, and emphasizes potential short and long-term side effects associated with extended intrathecal triamcinolone use.

## Introduction

Intrathecal administration of extended-release corticosteroids dates back to the 1950s and has been used as therapy for a number of neurologic conditions, including postherpetic neuralgia, pain management, and treatment of multiple sclerosis (MS) related spasticity (1–3). Originally, methylprednisolone acetate was used as a formulation, which, in addition to general known complications caused by lumbar puncture, was considered to be the cause of side effects such as arachnoiditis, aseptic meningitis, or generalized pachymeningitis (4). As a result, these risks combined with uncertain efficacy have led to a marked decline in the popularity of intrathecal steroid application (5–7), even after the introduction of novel steroid formulations such as triamcinolone (8). Moreover, therapeutic strategies, such as physiotherapy, application of botulinum toxin, oral or intrathecal baclofen, tizanidine, as well as cannabinoids have emerged as efficacious strategies with limited and controllable side effects (9, 10).

In this context, we here present another potential side effect of intrathecal steroid administration, namely repeated episodes of aseptic neutrophilic pleocytosis with up to 5,200 leukocytes/μl in the cerebrospinal fluid over a one-year period of triamcinolone treatment, alongside elevated cytokine release in the CSF. While the short and long-term sequelae of these alterations remain unclear, these observations underscore the need for further investigation of long-term intrathecal steroid application, and further strengthen the relevance of alternative treatment approaches for spasticity in neurological disorders.

## Case

A 76-year-old female patient with AQP-4 negative Neuromyelitis Optica Spectrum Disorder (NMOSD) first exhibited a spinal syndrome with predominantly right-sided tetraparesis in 2016. Immunomodulatory therapy with Rituximab was administered from June 2018 to December 2018. However, due to a lack of clinical improvement, the therapy was discontinued. To treat the persistent painful spastic symptoms, the patient underwent intrathecal triamcinolone therapy every 6 months starting in June 2019, with a cumulative dose of 80 mg over 2 days. Both the patient and treating physicians confirmed improvement in spasticity and mobility upon triamcinolone therapy.

**Figure 1:**
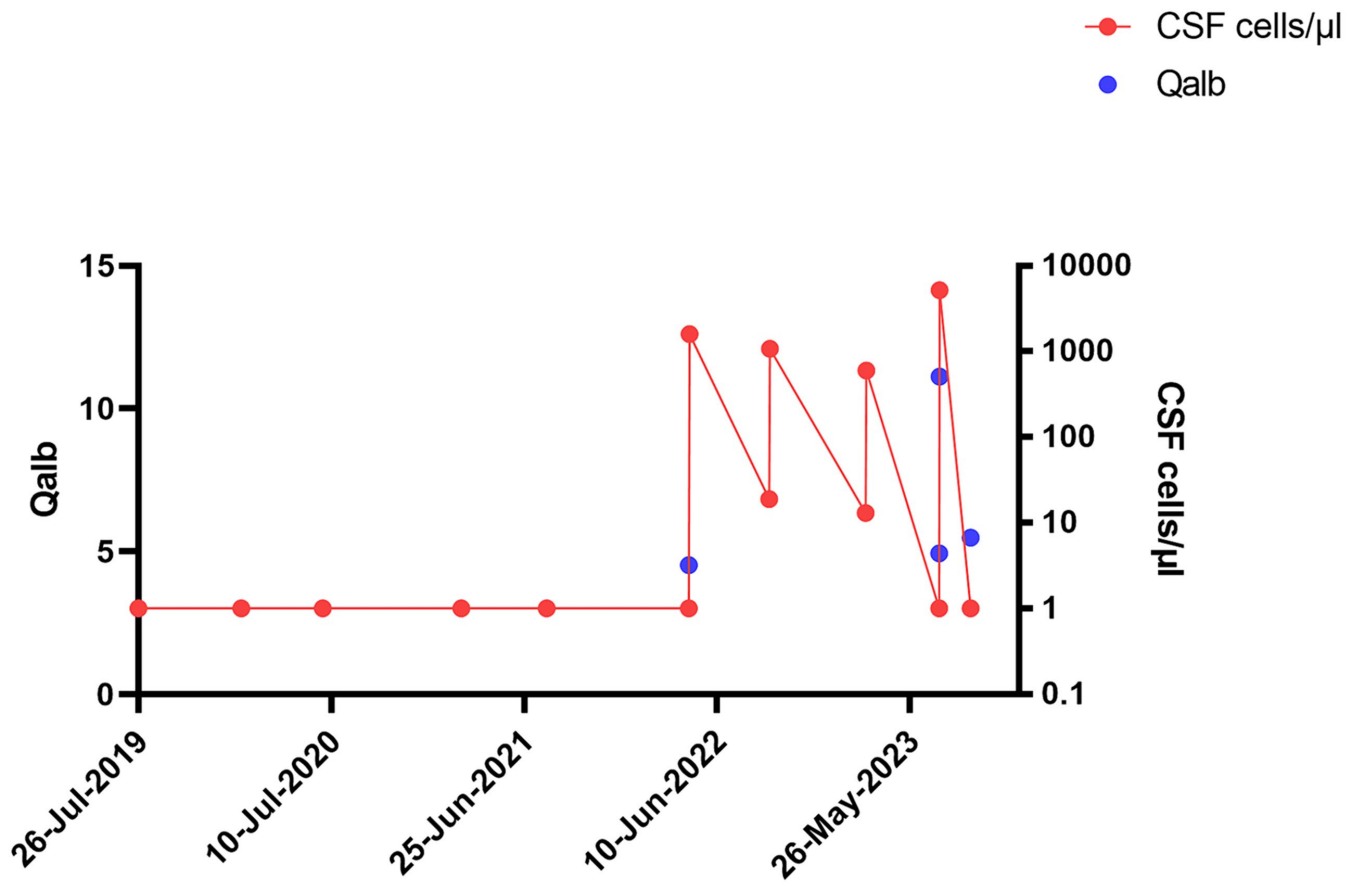
Longitudinal progression of cerebrospinal fluid (CSF) Qalbumin and cell count in index patient: Longitudinal progression of CSF Qalbumin (left y axis) and cell count (right y axis, cells/μl) in index patient.

**Figure 2:**
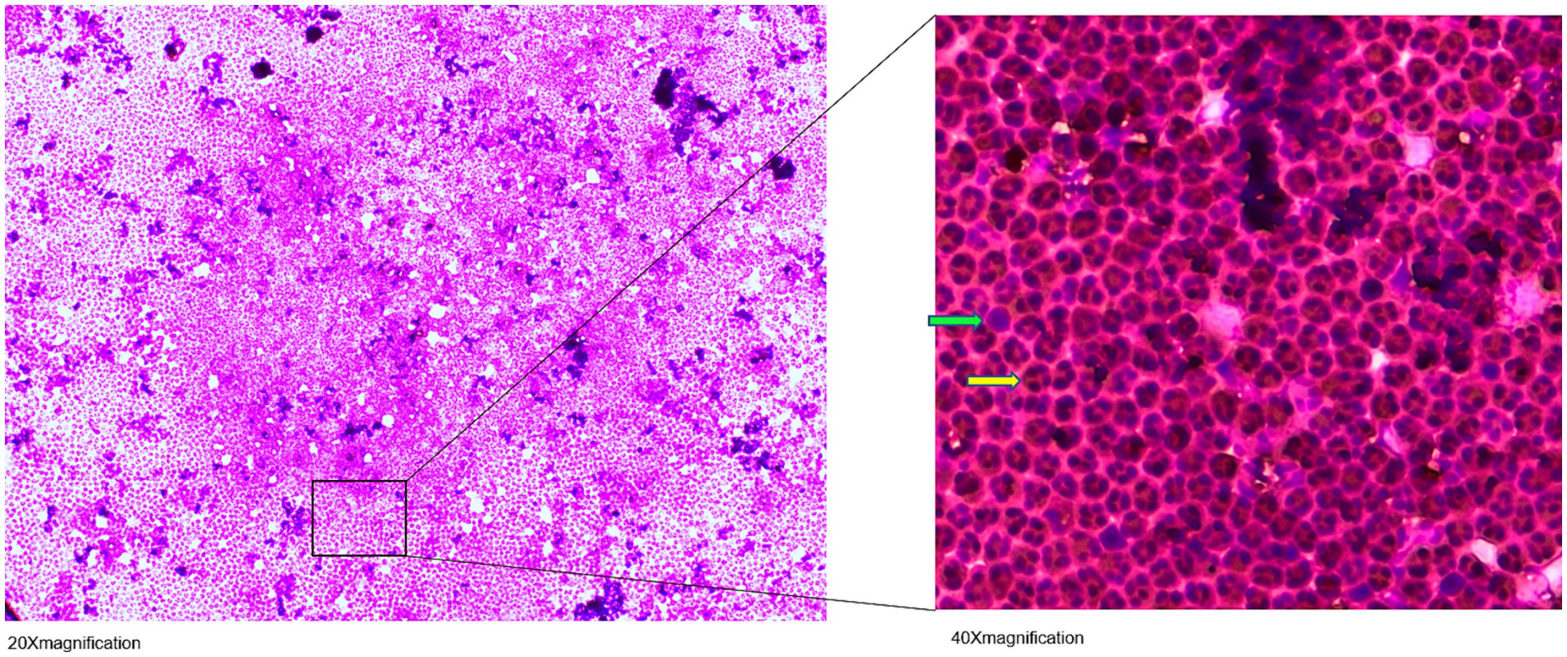
May-Grünwald-Giemsa stained cerebrospinal fluid (CSF) cell image: Visible are hypersegmented neutrophil granulocytes (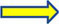), as well as occasional mononuclear cells (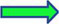).

**Figure 3:**
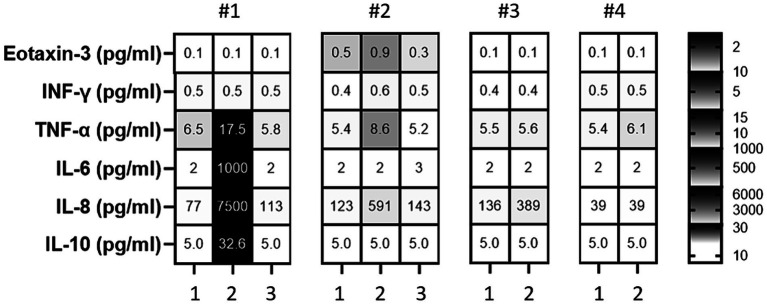
Cytokine profile in the cerebrospinal fluid (CSF) before intrathecal triamcinolone administration, 1 day after administration, and during follow-up. The heat map displays the concentrations of the cytokines eotaxin-3, interferon-γ (INF-γ), tumor necrosis factor-alpha (TNF-α), interleukin-6 (IL-6), interleukin-8 (IL-8), and interleukin-10 (IL-10) in the cerebrospinal fluid (CSF) of patients #1, #2, #3, #4 at two or three different time points: before (1) and 1 day after intrathecal triamcinolone administration (2), as well as after 2 months for patient #1 (3) and 3 months for patient #2 (3).

The patient tolerated the intrathecal triamcinolone injections well, with no clinical side effects or cerebrospinal fluid (CSF) abnormalities. However, the day after the fifth injection on 21 April 2022, CSF cell counts increased to 1,600/μl, consisting of 90% granulocytes. Subsequent lumbar punctures performed after the first administration on the following day and also at the beginning of the new cycle after 6 months continued to show consistently elevated cell counts (Figure 1). Spinal magnetic resonance imaging (MRI) on 15 July 2022, showed pre-existing myelon lesions at levels C4/5 to C6, but no signs of adhesions or arachnoiditis as a potential cause of the CSF findings. Also, clinical signs of meningitis were absent. Based on the beneficial clinical effects and the assumption of reactive pleocytosis due to steroid injection, intrathecal triamcinolone therapy was continued. However, after the 17th intrathecal administration of triamcinolone, a lumbar puncture on 19 July 2023, revealed a turbid CSF with excessively increased leukocytes (5,200/μL, 98% granulocytes), elevated albumin quotient (11.12) without intrathecal immunoglobulin synthesis (IgG/Q 6.8 [oligoclonal bands type IV]; IgA/Q 4.94, IgM/Q 3.63), a lactate concentration of 4.38 mmoL/L and a glucose concentration of 77 mg/dL. CSF granulocytes exhibited a hypersegmented nuclear pattern in cytology (Figure 2), indicative of an activation response. Despite the absence of elevated systemic infection parameters, the substantial increase in CSF leucocytes raised concern about a potential bacterial infection, and empiric treatment with ceftriaxone was initiated. Yet, negative infectious workup (broad-range 16S rRNA PCR analysis of CSF, CSF cultivation for bacteria and fungi, microscopic examination (Gram staining) and inhibition testing) led to assume triamcinolone-induced aseptic neutrophilic pleocytosis. To clarify the underlying pathomechanisms, comparative analyses of the cerebrospinal fluid (CSF) cytokine profile in this patient (#1), as well as three additional patients (#2, #3, #4) suffering from secondary progressive multiple sclerosis (MS), who had received an equivalent dose of intrathecal triamcinolone treatment for spasticity treatment, were performed. CSF samples were collected from patients #3 and #4 both before and 24 h after the administration of triamcinolone. Additionally, CSF samples were collected from patient #1 after a 2-month follow-up and from patient #2 after a 3-month follow-up. No changes in the CSF cell counts were observed in control patients. In-depth cytokine investigation detected a distinct pattern between index (#1) and control patients (##2, 3, 4). Specifically, we noted an increase in the levels of tumor necrosis factor-alpha (TNF-α), interleukin-6 (IL-6), interleukin-8 (IL-8), and interleukin-10 (IL-10) in the CSF of patient #1 after intrathecal triamcinolone administration, while no relevant changes were observed in the control patients. Conversely, no alterations were detected in the levels of interferon-γ (INF-γ) or eotaxin-3 (Figure 3).

In view of this inflammatory CSF syndrome, we discussed to pause further intrathecal administration of triamcinolone in order to assess the clinical course following the CSF pathology described above. Indeed, the patient did not exhibit additional clinical symptoms during the inpatient stay, especially no meningism or headache, and was discharged in unaltered general condition. After 8 weeks, we performed a follow-up diagnostic lumbar puncture, which showed normalization of all parameters pathological in the previous analyses (Figures 2, 3). Yet, in the light of the unclarity of the underlying causes for these CSF alterations as well as potential long-term side-effects, we discussed additional options for the treatment of the patient’s spasticity and finally decided together with the patient to discontinue intrathecal triamcinolone therapy.

Together, this case highlights a potential side effect of yet unclear short and long-term significance in the treatment of spasticity using intrathecal steroids. Future studies will need to address these and other long-term effects of intrathecal steroids on the one hand, while on the other hand additional established treatment strategies for spasticity gain increasing importance in the light of their limited side effects and long-term tolerability.

## Discussion

We here report a case of an asymptomatic neutrophilic pleocytosis after administration of triamcinolone characterized by an inflammatory CSF syndrome, which has not been described to this extent before. The diagnosis was made based on the abrupt increase in leukocyte count with increased albumin quotient and turbid CSF after the 17th intrathecal triamcinolone injection, after infectious meningitis was ruled out. The patient remained asymptomatic throughout the follow-up period.

Besides postlumbar puncture complications such as headache, hygromas, infections and tentorial herniation, lumbar punctures and intrathecal drug administration have been described to induce mild changes in various parameters of routine CSF diagnostics (11). First, CSF pleocytosis has been described as a reaction to the puncture *per se*, alongside instances of non-specific cell presence with a maximum count of up to 45 leukocytes/μL (12), most likely due to mechanical irritation. Second and more importantly, pleocytosis following intrathecal administration of medication has been described as a non-specific irritation to the chemical agent applied, with increases in cell numbers up to 453/μL (13). This phenomenon has been termed induced chemical meningitis, a non-specific reaction to the active substance used, the pathophysiological mechanism of which remains unclear. Direct meningeal irritation or a hypersensitivity reaction have being discussed in this context (6, 7, 14, 15). Indeed, previous reports have described cases of chemical meningitis with severe clinical manifestations, including fever and headache following intrathecal methylprednisolone acetate injection (4, 7, 14). These complications appeared to be associated with the molecular composition of methylprednisolone acetate, and might thus be less prominent under triamcinolone application. In these lines, animal research showed no evidence of neurotoxicity upon intrathecal administration of triamcinolone diacetate (16). Yet, the clinical and long-term implications of these CSF findings upon intrathecal triamcinolone application remain unclear to date. Indeed, even though no additional side effects of intrathecal triamcolone administration were described in larger studies (17–20), these observations must limit excessive use of triamcinolone to treat spasticity as long as long-term effects of these treatments remain unclear.

The pathophysiology underlying the abrupt and substantial increase in cell count and protein levels after the 17th intrathecal administration remains unclear. Alongside potential causative mechanical irritation caused by the lumbar punction and application of the medication, drug hypersensitivity reaction mediated by IgE antibodies needs to be taken into account as possible cause of these alterations (21). To provide clarification, an additional analysis of CSF cytokine profile was carried out. In this patient’s case, chemical meningitis was marked by a specific cytokine profile, with increased levels of IL-6, IL-8, and TNF-α, while INF-γ and eotaxin-3 levels remained unchanged. This suggested a different mechanism than a typical drug hypersensitivity reaction. Indeed, this reaction is most likely not be mediated by a classical allergic reaction, but might instead be exclusively triggered by proinflammatory cytokines, such as interleukin-6 (IL-6) and tumor necrosis factor-alpha (TNF-α), which might be directly induced by the administered intrathecal triamcinolone. These cytokines can act as chemotactic stimuli, attracting neutrophilic granulocytes into the cerebrospinal fluid (CSF) (22). This phenomenon can be compared to a cytokine release reaction (CRR) observed in cases where drugs such as rituximab or chemotherapeutic agents are administered systemically (21). However, it has not yet been described after intrathecal administration. In the context of meningitis, a notable distinction exists between infectious forms, such as viral or bacterial meningitis, and non-infectious cases. This difference is exemplified by the presence or absence of elevated IFN-γ levels. In infectious meningitis, there is typically an elevation of IFN-γ in response to the presence of pathogens (23). In contrast, in non-infectious cases, no notable increase in IFN-γ levels is observed, highlighting fundamental distinction between infectious-triggered meningitis and non-infectious causes.

Several additional limitations need to be taken into account when analyzing these observations: While the index patient suffered from seronegative NMOSD, the control patients without CSF alterations upon triamcinolone applications were diagnosed with secondary progressive multiple sclerosis. Thus, the pathophysiological differences of the underlying diseases *per se* may impact cytokine profiles and reactions elicited by the procedure. In these lines, the predominant IL-6 and neutrophil elevation in the CSF raises the question of disease reactivation in NMOSD, where IL-6 and neutrophils have been associated to relapse activity predominantly (24). Even though MRI scans detected no novel or contrast enhancing lesions, and clinical symptoms suggestive of acute disease activity remained absent, the potential side effect of disease activation is of concern, given the potential severity and therapeutic refractoriness of relapses in NMOSD. Finally, there are currently no clear guidelines on how to proceed in the event of chemical meningitis following intrathecal injections, as the underlying mechanisms remain unclear (25). Finally, potential late complications such as arachnoiditis are not assessed in the case presented here.

In this light, further research to clarify this phenomenon and its long-term implications is clearly warranted. Finally, additional anti-spasticity therapies have proven efficient and are thus first-line recommendations for the treatment of spasticity (9, 10). These therapies include physiotherapy, locally applied botulinum toxin, oral medications such as baclofen, tizanidine, or cannabinoids. Moreover, intrathecal continuous baclofen application has proven efficient, while severe side effects such as baclofen withdrawl syndrome must be taken into account. Together, these treatments need to be considered first-line options and can be escalated from non-invasive to increasing invasiveness, tailoring treatments to the needs of individual patients.

Together, this case describes an inflammatory CSF syndrome induced by intrathecal triamcinolone therapy most likely due to cytokine release syndrome resembling mechanisms. While it highlights a side-effect of intrathecal triamcinolone, the long-term relevance of which is yet unclear, it also emphasizes the need for caution in long-term application regimens of intrathecal steroids. It is imperative that this issue be discussed specifically in patients receiving intrathecal administration over an extended period of time, and that a strong focus on alternative treatment regimens be put, as long as the sequelae of these alterations are yet unknown. Future research should systematically examine the effects of leucocytes and the long-term consequences of the CSF inflammatory syndrome on patients to provide clinical guidelines for the management of chemical meningitis.

## Data availability statement

The raw data supporting the conclusions of this article will be made available by the authors, without undue reservation.

## Ethics statement

The studies involving humans were approved by Ethik-Kommission der Friedrich-Alexander-Universität Erlangen-Nürnberg. The studies were conducted in accordance with the local legislation and institutional requirements. The participants provided their written informed consent to participate in this study. Written informed consent was obtained from the individual(s) for the publication of any potentially identifiable images or data included in this article.

## Author contributions

TT: Writing – review & editing, Writing – original draft, Investigation, Conceptualization. JS: Writing – review & editing, Writing – original draft. VT: Writing – review & editing, Writing – original draft. MU: Writing – review & editing, Writing – original draft. SS: Writing – review & editing, Writing – original draft. DH: Writing – review & editing, Writing – original draft. VR: Conceptualization, Writing – review & editing, Writing – original draft.
